# A behavioral advantage for the face pareidolia illusion in peripheral vision

**DOI:** 10.1038/s41598-024-60892-z

**Published:** 2024-05-02

**Authors:** Blake W. Saurels, Natalie Peluso, Jessica Taubert

**Affiliations:** School of Psychology, The University of Queensland, St Lucia, Queensland Australia

**Keywords:** Face perception, Illusory faces, Continuous eye movements, Face categorization, Human behaviour, Object vision

## Abstract

Investigation of visual illusions helps us understand how we process visual information. For example, face pareidolia, the misperception of illusory faces in objects, could be used to understand how we process real faces. However, it remains unclear whether this illusion emerges from errors in face detection or from slower, cognitive processes. Here, our logic is straightforward; if examples of face pareidolia activate the mechanisms that rapidly detect faces in visual environments, then participants will look at objects more quickly when the objects also contain illusory faces. To test this hypothesis, we sampled continuous eye movements during a fast saccadic choice task—participants were required to select either faces or food items. During this task, pairs of stimuli were positioned close to the initial fixation point or further away, in the periphery. As expected, the participants were faster to look at face targets than food targets. Importantly, we also discovered an advantage for food items with illusory faces but, this advantage was limited to the peripheral condition. These findings are among the first to demonstrate that the face pareidolia illusion persists in the periphery and, thus, it is likely to be a consequence of erroneous face detection.

## Introduction

**Figure 1:**
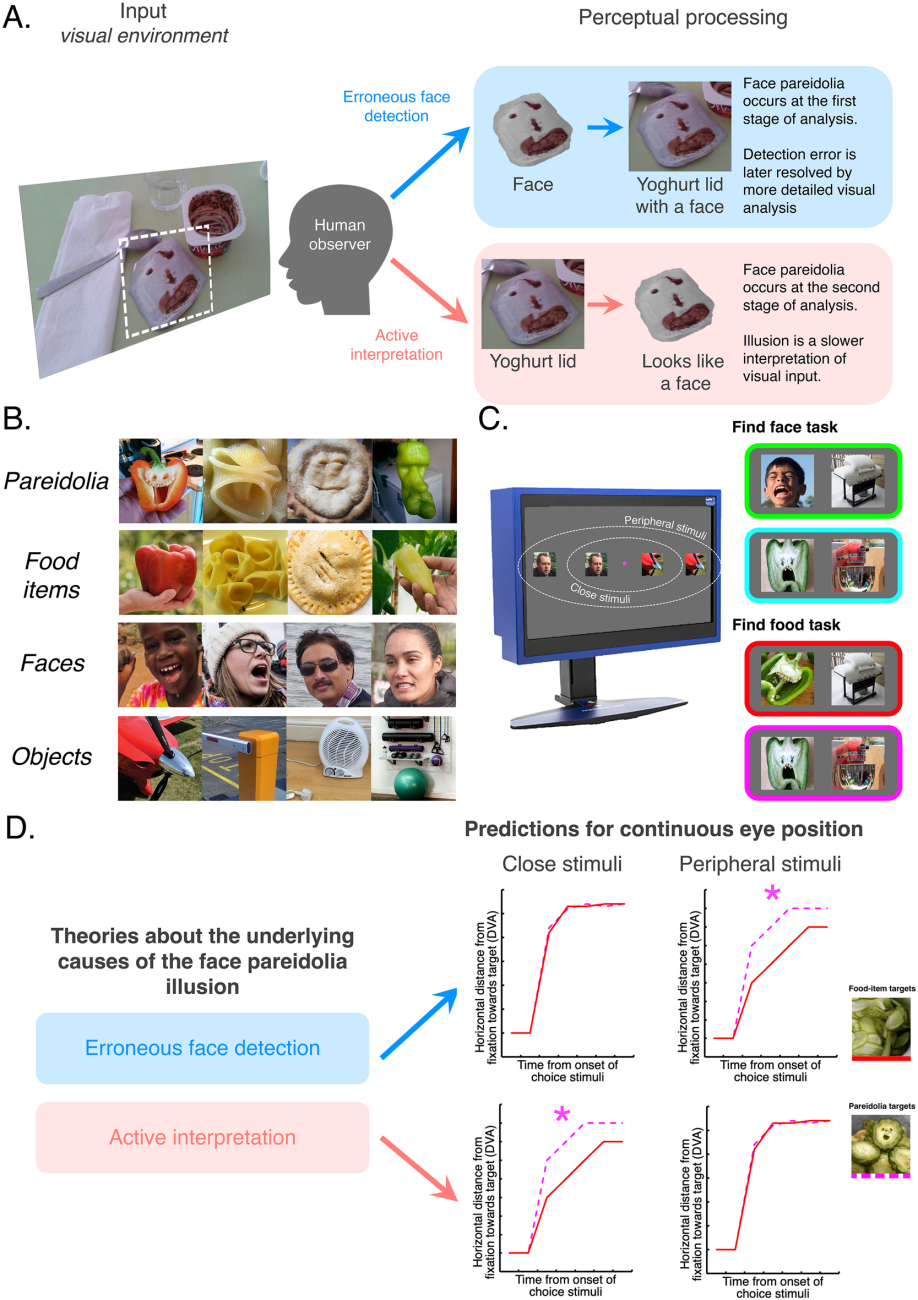
(**A**) Illustration of the competing theories currently used to explain our perception of *face pareidolia.* (**B**) Examples of visual stimuli used in the fast saccadic choice experiment. 40 Real faces and 40 examples of pareidolia were used as targets in the *find the face* task. The same examples of pareidolia and 40 matched food items were used as targets in the *find the food* task. In both tasks, the distractors were 80 images of inanimate, inedible man-made objects. The human face images were published under a CC-BY license and we have permission to publish here. Indeed, all stimuli used in the experiments are available at the Open Science Framework website: https://osf.io/2p6x7/. (**C**) On the *left* is a schematic of the participant’s screen showing the relative distance of choice stimuli in the close condition and the peripheral condition from the center of the screen (marked with a magenta dot). On the *right* are examples of the choice stimuli showing the four unique conditions (*green* = face target in the face task, *cyan* = pareidolia target in face task, *red* = food target in the food task, *magenta* = pareidolia target in the food task). In these examples the targets are shown on the left. During the experiment, target stimuli appeared on the left and the right an equal number of times. (**D**) Predicted biases in eye movement data in the find food task. Magenta asterisks flag conditions with predicted significant advantages for pareidolia over food items.

In cognitive neuroscience, there are two competing explanations for our experience of face pareidolia, a compelling visual illusion whereby we falsely perceive facial features in otherwise inanimate objects, like a piece of toast. One explanation is that when we are presented with a visual stimulus, we can actively interpret any ambiguity in visual input as being face-like^1–6^. From this perspective, the experience of face pareidolia requires fixation and attention to actively guide a decision-making process (Fig. 1A). Alternatively, there is evidence indicating that examples of face pareidolia drive activity in the parts of the primate brain responsible for reflexively, detecting faces in the larger visual environment^7–13^. Thus, from this perspective, the experience of face pareidolia is a symptom of a hypersensitivity to face-like patterns that we share with neonates and other primates^7,14–18^ (Fig. 1A). These two competing theories make different predictions about the perception of face pareidolia across the visual field; If the experience of face pareidolia reflects the active interpretation of visual ambiguity, then the perception of an illusory face will be dependent on foveal input, whereas if the experience of face pareidolia reflects an error of face detection, then the perception of an illusory face should be independent of foveal input.

At the behavioral level, most studies that have examined our response to the face pareidolia illusion have presented stimuli at fixation and asked participants to report how face-like the image looked^19–21^, where the illusory face was looking^22^, or how happy the illusory face appeared to be^23,24^. And, although a few studies have measured detection accuracy in the context of visual search^10^, continuous flash suppression^25^, and dot-probe^12^ paradigms there have been no attempts to determine whether the face-like advantages conferred by illusory facial features, survive in the periphery. Further, because previous studies of face pareidolia have often tasked participants with looking for faces or judging face-like qualities^9,19–24,26^, it remains unclear whether there are *spontaneous* behavioral advantages for examples of face pareidolia, when facial features are irrelevant to the task at hand. To address this gap in our understanding, we used a fast saccadic choice paradigm^27,28^ to determine whether the illusory facial features in ambient examples of face pareidolia summon eye movements to their location across two separate tasks. In one task, the participants were required to use their eye movements to select face targets as quickly as possible. In the other task, the participants were required to use their eye movements to select food targets as quickly as possible. Thus, we were able to employ the exact same examples of face pareidolia as targets in both tasks, i.e., food items *with* illusory facial features (see Fig. 1B), to facilitate a direct comparison across conditions. We also manipulated the distance of the choice stimuli from the central fixation point (Fig. 1C) because, if face pareidolia triggers the same processes that underlie the rapid detection and spontaneous prioritization of real faces, then any advantage for face pareidolia should increase with eccentricity^29–31^ (Fig. 1D). Alternatively, if the face pareidolia illusion relies on an active interpretation of high-fidelity visual input from the fovea then the any advantage for face pareidolia should decrease with eccentricity (Fig. 1D). Remarkably, the results uncovered a behavioral advantage for face pareidolia that was limited to when stimuli were presented further away from the initial fixation spot. This finding confirms that the face pareidolia illusion is spared in peripheral vision and suggests that illusory facial features that occur by chance in the environment are falsely detected as faces during initial, potentially pre-attentive, stages of perceptual processing.

## Results

We collected examples of face pareidolia that occurred by happenstance in food items, such as baked goods, vegetables and desserts (Fig. 1B). We then found a set of matching food items with no illusory facial features (Fig. 1B), together with a set of 40 ambient face images. These images all served as targets in the experiment. We note that pareidolia and food-items were matched primarily for semantic category (i.e., type of food), but visual and contextual properties were as similar as possible. The distractors for both tasks were drawn from a set of 80 inedible, man-made objects, such as appliances, vehicles and houses (Fig. 1B). Every participant (*N* = 24) completed two blocks of trials in a counterbalanced order while we continuously monitored their eye position (Fig. 1C; for more details see Methods). After a mandatory fixation period of a variable and unpredictable length, each trial presented participants with two choice stimuli, one on the left and one on the right. Choice stimuli were always equidistant from the center of the screen, but their distance differed across trials (Fig. 1C); in the close condition, choice stimuli were 6.5° from the central fixation point, whereas in the peripheral condition, choice stimuli were 10.8° from the central fixation point (see Methods). The decision to use these two distances from the central fixation point was informed by previous literature^27,28,32,33^ and the constraints of the equipment (i.e., the width of the monitor and the sensitivity of the eye-tracker).

### Main results

**Figure 2:**
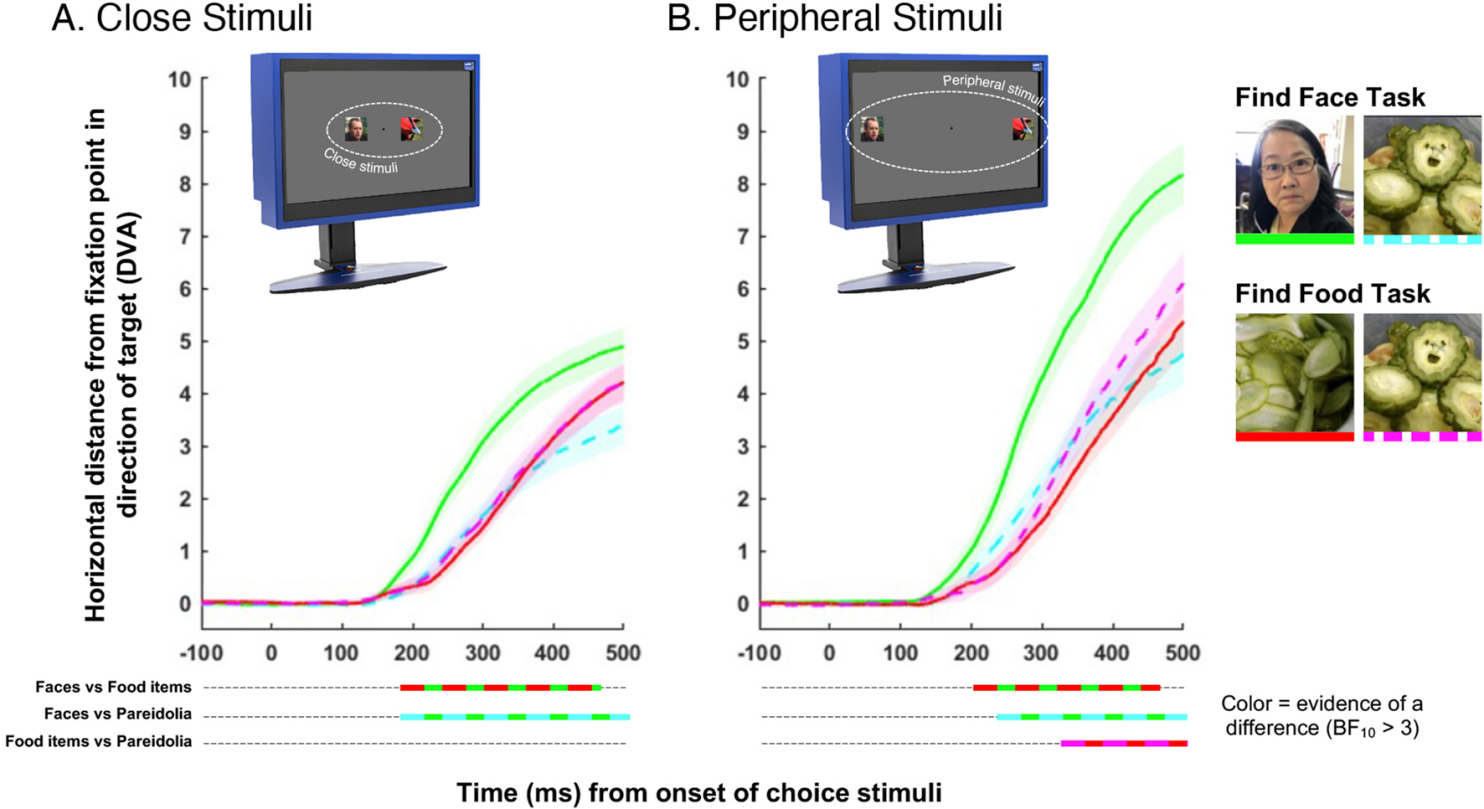
(**A**) Average horizontal eye position in degrees of visual angle (DVA), when target stimuli were presented close to fixation as a function of time from the onset of choice stimuli (transparent regions =  ± *SE*). Line color represents the four unique conditions. The time course of significant pairwise differences are provided underneath the* x*-axis. (**B**) Average horizontal eye position when target stimuli were presented in the periphery. Same conventions as (A).

Our results replicated the main finding from previous fast saccadic choice experiments^27,28,30,32^—we found that participants moved their eyes towards face targets faster than they were able to do so for food targets. Bayesian factor analysis revealed evidence [BF_10_ > 3] of an advantage for faces over food items from 190-ms after image onset in close trials, and 206-ms in peripheral trials (Fig. 2A, B, green vs red lines). Unlike cars or flowers, food items are arguably more comparable to faces in terms of biological imperatives. For example, being able to find social agents and sources of nutrition are both central to our survival. Further, recent neuroimaging evidence has suggested that food items are processed in category-selective areas adjacent to those responsible for processing faces^34^. However, despite this similarity, we found evidence that we find pictures of faces faster than pictures of food.

In line with previous findings, we also found an advantage for real face targets over pareidolia targets when participants were looking for faces, which was evident from 197-ms in close trials, and 230-ms in peripheral trials (Fig. 2A, B, green vs cyan lines). This is likely because human faces differ from food items and other objects on many visual dimensions (for e.g., color, curvilinearity). These differences provide low-level cues that can be exploited to locate human face targets^35^. The significance of the current findings, however, is that objects with illusory faces have an advantage over objects without illusory faces. In this experiment, care was taken to match pareidolia targets with food item targets in terms of semantic category and visual appearance (see Fig. 1B). This nested design has been exploited in previous studies of ambient face pareidolia because it means any differences in the behavioral or neural correlates are driven by the presence of illusory facial features^7,9–11,14,19,21,23,24,36^. Thus, the current results suggest that the advantage for faces in the fast saccadic choice paradigm is supported, at least in part, but the visual information that real and illusory faces share in common.

More importantly, we found that the advantage for face pareidolia was only evident in the peripheral condition (Fig. 2B, magenta vs red lines). In contrast, when the choice stimuli were positioned closer to fixation, there was no evidence of an advantage for face pareidolia (Fig. 2A, magenta vs red lines). These are key, complimentary findings because they indicate that the face pareidolia targets used in this experiment were not merely more salient and easier to detect than the non-face, food targets. Instead, the advantage for examples of face pareidolia over matched food items only occurred when participants had lower visual acuity during the initial fixation period and limited access to the visual details associated with the choice stimuli. When eccentricity is increased, it has been shown that biases in fast saccadic choice tasks reflect coarse spatial content and global face-like patterns^32^. Therefore, finding an advantage for face pareidolia that is limited to the peripheral condition not only indicates that the illusion is independent of foveal vision, it suggests that we are more prone to erroneously detecting face-like patterns in the periphery and may require fixation to resolve these errors^9^.

### Item-based analyses

**Figure 3:**
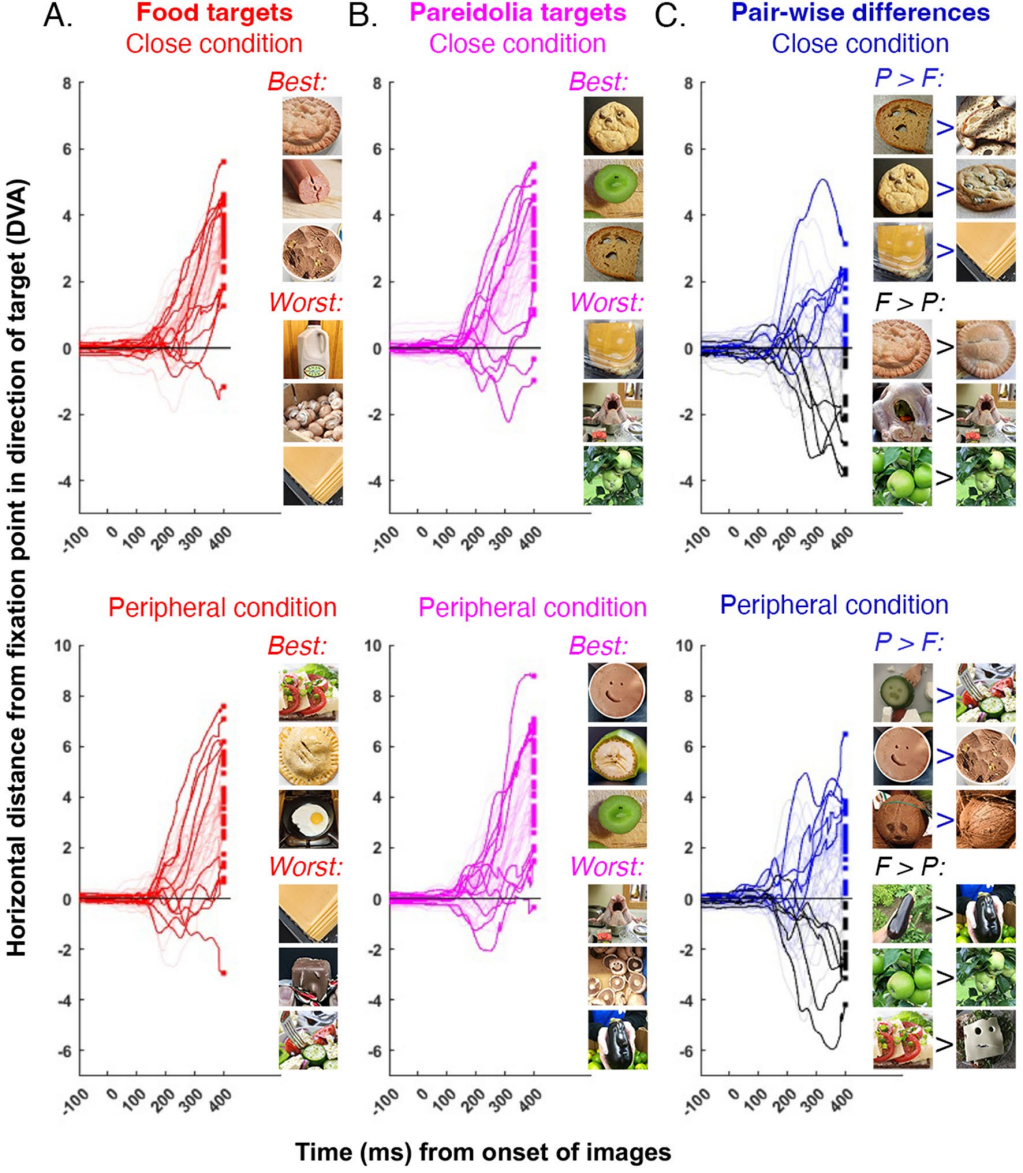
Results of the item-based analysis. All data taken from trials where the task was to find the food (**A**). *Top,* average horizontal eye position in degrees of visual angle, for every food target in the close condition as a function of time from the onset of choice stimuli. Food targets represented as separate red lines (bold lines emphasize the five “best” and the five “worst” performing target stimuli at the 350-ms time point, i.e., the food items that were detected the fastest and the slowest). Also included are the three best (i.e., drove the fastest detection response) and the three worst (i.e., drove the slowest detection response) food targets. *Bottom,* average horizontal eye position for every food target in the peripheral condition as a function of time from the onset of choice stimuli. Same conventions as above. (**B**) *Top,* average horizontal eye position in degrees of visual angle, for every pareidolia target in the close condition as a function of time from the onset of choice stimuli. Pareidolia targets represented as separate magenta lines (bold lines emphasize the five “best” and five “worst” performing target stimuli at the 350-ms time point). Also included are the three best (i.e., drove the fastest detection response) and the three worst (i.e., drove the slowest detection response) pareidolia targets. *Bottom,* average horizontal eye position for every pareidolia target in the peripheral condition as a function of time from the onset of choice stimuli. Same conventions as above. (**C**) *Top,* we examined the advantage for pareidolia for every matched pair of stimuli in the close condition subtracting the average horizontal eye position for a pareidolia target from the average horizontal eye position for the corresponding food target. Dark lines emphasize the five stimulus pairs with the largest positive difference (i.e., pareidolia targets > food targets) and the five stimulus pairs with the largest negative difference (i.e., pareidolia targets < food targets). Also included are the three pairs of stimuli that the strongest bias towards pareidolia targets, and the three pairs of stimuli that the strongest bias towards food targets. *Bottom,* we examined the advantage for pareidolia for every matched pair of stimuli in the peripheral condition. Same conventions as above.

Item-based analyses were performed for data from the “*find the food*” trials (see Fig. 3A for food targets and Fig. 3B for pareidolia targets). This approach revealed that some targets were more effective than others, but nonetheless there was a similar range of behaviourial responses evoked by food and pareidolia targets. Next, we took advantage of the matched pairs in the stimulus design by comparing discrete pareidolia targets to their corresponding non-face, food targets (see Fig. 1B). These stimulus pairs were matched for sematic and visual characteristics^7,9–11,14^. Figure 3C shows that the size of the advantage for examples of face pareidolia over matched food items was not uniform across all stimulus pairs. Collectively, these observations suggest that some illusory faces recruit face detection mechanisms more successfully than others. Why would this be the case?

A number of researchers have previously described variation in the strength of the illusory percept in examples of face pareidolia^19,21,37^ and although there have been numerous attempts to determine the source of this variation using models of low-level saliency, curvature and dominant spatial structure^9,19,37^ as well as deep neural networks trained on large image sets^9,19^, none of these models have been able to predict face pareidolia illusion, perhaps owning to their high-degree of visual variability among examples of face pareidolia. For example, face pareidolia can occur on the side of a building, in a multi-colored burger being held in two hands or a small, green button. Nonetheless, it remains the case that some collection of low- and/or mid-level properties must determine which specific examples of face pareidolia will be more visible and resistant to changes in eccentricity. For example, perhaps when illusory facial features are carried by low spatial frequencies or high contrast, the illusion is more likely to survive in the periphery and bias behavior in the fast saccadic choice task^28,32,38,39^. We note that the examples of face pareidolia that drove the strongest behavioral responses in the close distance condition were not the same examples of face pareidolia that drove the strongest behavioral responses in the peripheral distance condition (see Fig. 3). This further indicates that the viewing behavior of the participants were driven by different visual properties and features depending on the distance of the choice stimuli from the initial fixation spot. More research is needed to understand the visual features that guide eye movements when stimuli are not presented in foveal, or near-foveal, vision (see^40^). Even so, regardless of what visual properties promote the face pareidolia illusion, and whether these change across the visual field, overall our results reveal that ambient examples of face pareidolia bias viewing behavior towards peripheral locations.

## Discussion

Ambient examples of face pareidolia are emerging as an important tool for probing face detection^7–12,14,20,23^ and its dysfunction^13,25,26,37,41^. But debate about the neural processes that give rise to face pareidolia has stalled scientific progress. These results provide much-needed empirical support for the hypothesis that examples of face pareidolia trigger the mechanisms responsible for rapid face detection, by revealing that the face pareidolia illusion survives in peripheral vision (Fig. 1A). Importantly, these results demonstrate that, like monkeys^7,14^, humans are equipped with neural mechanisms that spontaneously prioritize the processing of faces by detecting coarse face-like patterns in the visual field and driving eye movements towards their spatial location.

In the comparative literature, biases towards face pareidolia in eye tracking studies have been used to identify the neural mechanisms underlying face detection and prioritization. For example, it has been shown that while typically developing rhesus macaques preferentially look at examples of face pareidolia^14^, and illusory facial features^40,42^, in free viewing tasks, this implicit, untrained, behavior is eliminated following the bilateral removal of the amygdala^7^. This is further supported by neuroimaging studies showing that examples of face pareidolia activate the amygdala in the primate brain^11,13^. However, until now, it was unclear whether the viewing behavior of human adults is also biased towards face pareidolia. The current experiment addressed this knowledge gap; the observation that the presence of illusory faces spontaneously biased eye movements when human participants were looking for food, helps bridge the gap between studies of human and nonhuman primates and validates the use of the macaque model to understand visual processing in the human brain. Therefore, an implication that directly follows from the current results is that the human amygdala, a face-responsive subcortical region^43^, might play a causal role in detecting faces in the visual environment and guiding eye movements towards their location. Although studies have started to look at the impact of amygdala disruption on eye movements towards faces in human participants^44^, more research is needed to determine whether amygdala activity is sufficient for biasing oculomotor behavior towards faces or, alternatively, whether this bias is the consequence of interactions between the amygdala and other brain regions and circuits^40^.

In this study, we used a fast saccadic choice task to measure continuous eye position as the dependent measure. This afforded us more granularity per trial than bespoke detection tasks that measure accuracy and reaction time. However, the key advantage of this approach is that we avoided the limitations typically associated with forced choice tasks, such as the influence of decisional criteria^45^. This limitation is likely amplified when presenting illusory stimuli, such as examples of face pareidolia, because the ground truth is at odds with what researchers might consider the “correct” or desired behavior. In other words, if you ask participants to select faces, or judge when a stimulus has facial attributes, then an important consideration is that examples of face pareidolia are inanimate objects that merely resemble faces. Therefore, the results of explicit, forced choice tasks will often depend on contextual factors, including how participants were instructed and whether they were given guidance about how to respond to examples of face pareidolia. These dependencies likely explain some inconsistencies emerging in literature^46–48^. For example, one behaviourial study of face pareidolia that used visual search reported an advantage for illusory faces over matched non-face objects^10^, and another found a limited advantage for illusory faces^49^. Here, we found an implicit behaviourial advantage for pareidolia over food targets in continuous eye movement data, even though participants were instructed to find food items, and thus there was no mention faces.

Another complication that has constrained previous studies involving the face pareidolia illusion is that it is difficult to interpret the differences evoked by real and illusory faces. Because ambient examples of face pareidolia only share limited visual information with real faces^20,37^, the expectation is that illusory faces will not elicit the same response as real faces. For example, in the current study, when we compared continuous eye movements across target types in the *find the face* condition, we found that participants looked at real face targets faster than pareidolia targets (Fig. 2). This finding is consistent with other behavioral^10,12,20^ and neural^9,11,13,36^ studies reporting that illusory faces drive approximately half of the response of real faces, and it suggests that when participants are explicitly instructed to find faces, real faces elicit stronger orienting responses than illusory faces. However, it is difficult to infer from the results of the *find the face* condition, whether the pareidolia targets conferred a face-like advantage or not (Fig. 2). Instead, all we know from the results of the *find the face* condition alone is that the average orienting response towards real and illusory faces is different. This highlights the importance of measuring behavior towards examples of face pareidolia, when the presence of illusory facial features are of no consequence.

In this study we designed a parallel task where participants completed the same fast saccadic choice paradigm, but instead of looking for faces, they were looking for food. Thus, in addition to measuring behavior towards face pareidolia without instructing the participants to look for facial features or measuring explicit decisions, this dual-task design circumvented the limitations associated with comparing behavioral responses to real and illusory faces. Our results revealed that when participants were required to *find the food* there was an advantage for food items with illusory facial features (i.e., pareidolia targets) over food items without illusory facial features (i.e., food targets). Importantly, this advantage was only evident when stimuli were presented in the periphery—when stimuli were presented closer to fixation there was no such advantage (Fig. 2). This is an important complimentary finding because it rules out the notion that the chosen pareidolia targets were inadvertently more visually salient than the chosen non-face food targets. Therefore, overall, these findings demonstrate that the spontaneous bias towards pareidolia targets in viewing behavior is dependent on peripheral presentation.

An outstanding question is what visual attributes drove the advantage for face pareidolia over food targets in the peripheral condition? Researchers have attempted to address this question in previous research, but no satisfying answer is available at present. One possibility, originally posited by Epihova and colleagues (2022), is that images containing face pareidolia have spectral and spatial properties that match faces^37^. This global structure, sometimes referred to as face template or bar code^50–52^, may be immune to peripheral shifts. However, we note that other attempts to explain behaviourial responses to face pareidolia using the same spectral and spatial properties^53^ have been unsuccessful^9,19^. Another study has indicated that the presence of specific illusory facial features predicts behavior towards examples of face pareidolia^20^. Omer et al. (2019) discovered that the removal of illusory eyes resulted in examples of face pareidolia being rated as less face-like than when the illusory mouth was removed. It follows that the primate brain may accumulate evidence for “resemblance to a face” judgements based on how many face-like features are present, with a particular emphasis on the presence of illusory eyes^20,22^. Interestingly, this theory is consistent with eye tracking studies in macaques showing that the illusory eyes, in examples of face pareidolia, attract a disproportionate amount of attention^14,40,42^. However, in the current study, the illusory eyes were available to participants in both the close and peripheral conditions. Thus, if their presence alone was sufficient to drive a bias in oculomotor behavior, we should have found advantages for pareidolia over food targets irrespective of the distance condition, but this was not the case (see Fig. 2). Therefore, more research is needed to distil the visual attributes underlying the spontaneous detection and prioritization of face pareidolia in the periphery.

We note that while the current results are generally consistent with the view that ambient examples of face pareidolia are erroneously detected as faces during the early stages of perceptual processing (Fig. 1A), it remains possible that this processing occurs outside of our awareness and is independent of conscious perception. For example, it has been shown that blindsight patients will preferentially orient their eyes towards the spatial location of face stimuli even when they do not report perceiving faces^54–57^. This might help explain mixed findings in the comparative literature; while macaque monkeys preferentially orient their eyes towards examples of face pareidolia in eye tracking studies^7,14,40^, it was recently reported that members of the same species that are conditioned to select faces, do not categorize ambient examples of face pareidolia as “faces”^58^. This implies that, while the macaque brain might initially mistake objects with illusory faces as being face-like, later processes decide that examples of face pareidolia are objects, not faces. Therefore, it remains possible that our awareness of the face pareidolia illusion, as humans, might be dependent on slower cognitive processes, beyond putative face detection^2,4–6^. It follows that both neural models presented in Fig. 1A could be valid, describing two, independent, processing stages that are necessary to experience the face pareidolia illusion. This underscores the need for future research to determine whether implicit and explicit behavioral makers of the face pareidolia illusion have different neural sources.

## Methods

### Participants

We recruited 27 volunteers from subject pool regulated and maintained by the University of Queensland. Three participants were unable to successfully complete the 13-point calibration and, thus, we analyzed the data from 24 participants of which 18 were female (on average these participants were 26 years old, ± 7.6 years), and 6 were male (on average these participants were 23 years old, ± 1.75 years). All participants had normal or corrected to normal vision. All procedures were approved by The University of Queensland Low and Negligible Risk Ethics Sub-Committee and were performed in accordance with the relevant guidelines and regulations, including the Declaration of Helsinki. Participation was voluntary and anonymous, and every participant provided informed consent.

### Visual stimuli

For use as targets in this experiment we selected 120 ambient images of human faces and food items from openly available stimulus sets used in previous studies^9,19,23^. Forty of the targets were examples of face pareidolia that occurred by happenstance in food items, such as baked goods, sliced vegetables and desserts (see Fig. 1B). For every example of face pareidolia, we found a matched, non-face, food item, i.e., the same type of food with no obvious illusory facial structure (see Fig. 1B). The 40 real face targets depicted humans that vary in age, gender, and emotional state. The 80 distractors were drawn from a set of photographs taken of inedible, man-made objects (Fig. 1B). Authors J.T. and B.W.S. determined that these 80 images depicted inanimate, man-made objects that were not edible. Examples of these kinds of objects include, vehicles, machines, gym equipment and tools (see Fig. 1B). All images were cropped square and resized (400 × 400 pixels) but no other manipulations were made. Cropping and resizing were completed in Photoshop 2020 (v. 21.2.0) and MATLAB R2020a.

### Experimental apparatus and procedure

In this study we employed a fast saccadic choice experiment^27,28^. The repeated measures design included two Block Tasks (*find the face* and *find the food*) with 2 Target Types (real and pareidolia) and 2 Distance conditions (close and peripheral) per task. Participants were seated 72 cm from the screen (ViewPixx CRT replacement monitor, 60 Hz refresh rate; VPixx Technologies). Before the experiment began, a 13-point calibration was performed using TrackPixx3 (2 kHz binocular eye tracker; VPixx Technologies) standard routines for eye tracking setup.

Every participant completed two blocks of trials in a counterbalanced order while we monitored their eye movements (Fig. 1C). In one block of trials the task was to *find the face*. For half of these trials, the target was a real face, and for the other half, the target was an example of face pareidolia (Fig. 1C). In the other block of trials, the task for participants was to *find the food*. Again, for half of these trials the targets were ordinary food items, for the other half, the targets were examples of face pareidolia. Thus, the exact same examples of face pareidolia were used as targets in both task blocks. At the beginning of each block of trials, a calibration check was conducted by measuring the gaze position of the participants when looking at 5 points of interest, including fixation and the center of each of the 4 possible image positions, which was later used to correct eye tracking data.

Every trial began with a 1000-ms inter-trial interval period during which a progression circle was presented to the participant indicating how many trials were left in the block. The progression circle was then replaced with a black fixation cross (0.43-dva in height and width) at the center of the screen for a period of time that varied between 800 and 1600-ms, followed by a 200-ms blank screen that served as a cue for the appearance of images. The variable fixation period was to reduce anticipatory eye movements. Next the images were presented in pairs for 400-ms and were quickly replaced with white crosses the same size as the fixation point. Participants were instructed to fixate on the initial fixation cross until it disappeared and then look to look at the location of the target image as quickly as possible. They were warned that that images would quickly disappear. The crosses marking the position of the two choice stimuli remained visible for 600-ms.

During the initial fixation period at the beginning of a trial, a fixation window with a radius of 1.3-dva was superimposed over the central fixation cross. If the participants gaze went outside of this window, even briefly, a warning screen was presented for 3 s, stating "You broke fixation on that trial. Please try to fixate on the black cross while it is on the screen." If during the course of the trial the participant realized they had made a mistake and initially looked in the direction of the wrong image, they were instructed to correct this mistake and look at the position of the correct image as soon as possible.

Every target stimulus was shown 4 times during the experiment; twice in the *close stimuli* condition (once on the left and once on the right) and twice in the *peripheral stimuli* condition (once on the left and once on the right). Therefore, there were a total of 320 trials per block. There was a 3–5 min break between blocks, followed by the ‘calibration check’ step.

### Raw eye signal preprocessing

The TrackPixx data buffer was initiated before each fixation period, ensuring that data was being captured and stored accurately. After the trial was complete, the data buffer was stopped, and the new trial data was pulled out for further analysis. At the beginning of each subsection of the trial sequence, a time tag was taken to index against the buffer times in the data sorting stage, allowing for precise synchronization of the data with the events of the trial. The data format was in pixels from the center of the screen, for both the *x*- and *y*-axes, for each eye. This format allowed for accurate recording of the gaze position.

To analyze these data, the eye gaze data from the fixation period and from the remainder of the trial were partitioned out of the original buffer data using the time tags recorded during each subsection of the trial sequence. Additionally, the data from the calibration check step at the start of each block was also extracted from the original buffer data.

The data from the calibration check step were used to correct the position of all data by the amount of error from the central fixation point that each participant had on each block, separately for each eye, along both the *x*- and *y*-axes. Each trial was then checked for deviations from fixation. From approximately 200-ms into each fixation period until the end, each individual data point was checked to see which eye had the least error from fixation. If the eye with the least error from fixation exceeded 1.3° of visual angle (DVA) (3 times the diameter of the fixation cross), then this was considered a deviation from fixation. If this occurred for more than 5 ms total, then the trial was considered a fixation deviation trial and was excluded from analysis. This occurred on approximately 14% of trials (the range across participants was < 1–37%).

We tallied which eye had the least error on each trial (that was not a fixation deviation trial). This information was used in combination with a visual inspection of the eye tracking performance at the time of calibration to determine the best eye to use. This eye was then used for all analyses conducted on the data from that task block. After this, any remaining data point (within trials) where the coordinates for eye gaze exceeded 15.1 DVA along the horizontal axis and 6.5 DVA along the vertical axis were removed. These points were well outside the range that test stimuli were shown in and have been associated with distractions, among other factors. Finally, the trials were sorted by conditions, including Task Block (*find the face* vs *find the food*), Target Type (*real* vs *pareidolia*), and Distance (*close stimuli* vs *peripheral stimuli*), for further analysis (see Fig. 1C).

### Horizontal eye position

To analyze viewing behavior during trials, the time resolved horizontal eye position data from every trial was computed relative to the target. As such, when the horizontal eye position is positively signed, this indicates that the participant was looking towards the target, whereas when the horizontal eye position is negatively signed, this indicates that the participant was looking towards the distractor. Additionally, the magnitude of the horizontal eye position indicates the distance in DVA from the central fixation spot. Thus, the larger the horizontal eye position, the further away from the center of the screen the participant was looking. For every participant, we computed the average horizontal eye position as a function of time, for all of the trials in each of the unique experimental conditions; there were 8 experimental conditions in total (2 Task Blocks × 2 Target Types × 2 Distance conditions). Finally, we averaged the data across all participants. These results are visualised in Fig. 2.

Bayesian factor analysis was then conducted to determine if, and when in the trial time course relative to the onset of the choice stimuli, there was evidence that average viewing behaviour differed across conditions. For every time point (in 1 ms time windows) we tested the following comparisons of interest: Real face targets (*find the face* task) vs real food targets (*find the food* task), real face targets (*find the face* task) vs pareidolia targets (*find the face* task), and real food targets (*find the food* task) vs pareidolia targets (*find the food* task). Significant deviations in horizontal eye position were identified using the following rule: BF_10_ greater than or equal to 3 (i.e., moderate evidence for the alternative hypothesis). All time points where there was evidence of a significant pair-wise difference in average horizontal eye position are highlighted in Figs. 2A (for *close stimuli* conditions) and **2B** (for *peripheral stimuli* conditions). For Fig. 3 the same Bayesian factor analysis was conducted on difference scores.

## Data Availability

Upon acceptance the original experimental stimuli (full resolution) and the datasets generated and analysed during the current study will be publicly available via the Open Science Framework: https://osf.io/2p6x7/.
